# Young age: an independent risk factor for disease-free survival in women with operable breast cancer

**DOI:** 10.1186/1471-2407-4-82

**Published:** 2004-11-17

**Authors:** Wonshik Han, Seok Won Kim, In Ae Park, Daehee Kang, Sung-Won Kim, Yeo-Kyu Youn, Seung Keun Oh, Kuk Jin Choe, Dong-Young Noh

**Affiliations:** 1 Department of Surgery, Seoul National University College of Medicine, Seoul 110-744, Korea; 2 Department of Pathology, Seoul National University College of Medicine, Seoul 110-744, Korea; 3 Department of Preventive Medicine, Seoul National University College of Medicine, Seoul 110-744, Korea; 4 Cancer Research Institute, Seoul National University College of Medicine, Seoul 110-744, Korea

## Abstract

**Background:**

The incidence of breast cancer in young women (age < 35) is low. The biology of the disease in this age group is poorly understood, and there are conflicting data regarding the prognosis for these women compared to older patients.

**Methods:**

We retrospectively analyzed 2040 consecutive primary invasive breast cancer patients who underwent surgical procedures at our institution between 1990 and 1999. The younger age group was defined as patients aged <35 years at the time of diagnosis. The clinicopathological characteristics and treatment outcomes were compared between younger and older age groups.

**Results:**

A total of 256 (12.5%) patients were aged <35. There was a significantly higher incidence of nuclear grade 3 and medullary histological-type tumors in younger patients compared to older patients. Axillary lymph node status, T stage, histological grade, c-erbB2 expression and estrogen receptor status did not differ significantly between the two age groups. Younger patients had a greater probability of recurrence and death at all time periods. Although there was no significant difference in disease-free survival between the two age groups in lymph node-negative patients, the younger group showed worse prognosis among lymph node-positive patients (p < 0.001). In multivariate analysis, young age remained a significant predictor of recurrence (p = 0.010).

**Conclusion:**

Young age (<35) is an independent risk factor for relapse in operable breast cancer patients.

## Background

Breast cancer is relatively rare in women less than 35 years of age, with this group accounting for less than 4% of the total number of breast cancer cases diagnosed in Western countries [1,2]. Despite the disease being relatively uncommon, it has a severe negative effect on the patients and their families.

It remains controversial whether young age at diagnosis is an adverse prognostic factor in primary breast cancer. While some studies have found that younger patients have worse clinical outcomes than older patients [3-7], others report younger patients have a more favorable prognosis, or that there is no relationship between outcome and age [8-10]. Various explanations have been given for these conflicting results, including small numbers of patients comprising the study population, differences in patient selection criteria and differences in the age groupings used in the analyses. Moreover, it has long been debated whether breast cancer diagnosed at a young age is a clinically and etiologically distinct disease from breast cancer diagnosed later in life. Some researchers reported that tumors in younger women were of higher grade, higher proliferation fraction, had more vascular invasion, and expressed fewer estrogen and progesterone receptors compared to tumors in older women [11-14]. It is important for clinicians to clarify the existing controversy as to whether aggressive treatment for young women with breast cancer is justified.

Breast cancer is the most frequent cancer in Korean women and its incidence is increasing [15]. Breast cancer in young Korean women is a serious problem, with the proportion of young age-onset breast cancer much higher than in western countries. According to the 2002 annual report of the Korean central cancer registry, breast cancers that developed before the age of 35 comprised 9.5% of all breast cancers [16].

The aim of the present study was to retrospectively investigate clinicopathological characteristics and prognosis in a large, ethnically homogeneous group of young breast cancer patients (less than 35 years old) treated with the same strategy at a single institution.

## Methods

A retrospective review was performed of the medical records of all consecutive primary invasive breast cancer patients (not including phyllodes tumor) undergoing curative surgery in the Department of Surgery, Seoul National University Hospital between January 1990 and December 1999. Patients with distant metastasis detected at the time of surgery or within 4 months of surgery were excluded. Those patients whose surgical margins were positive for malignancy were also excluded. Patients' records were reviewed for the following: age of onset, family history of breast cancer in 1st or 2nd degree relatives, histological type of cancer, tumor size in pathology reviews, axillary lymph node status, histological grade (HG: Scarff-Bloom-Richardson classification), nuclear grade (NG: Black's nuclear grade), type of surgical procedure and adjuvant therapy administered. Disease was staged according to the American Joint Committee of Cancer (AJCC) system [17]. The 'younger' group was defined as patients less than 35 years old at the time of breast cancer diagnosis. Expression of immunohistochemical tumor markers such as estrogen receptor (ER), progesterone receptor (PR) and c-erbB2 were determined in over 70% of cases. The expression was determined in assays performed immediately after surgery for each case. The primary antibodies for ER (DAKO, Glostrup, Denmark), PR (DAKO, Glostrup, Denmark) and c-erbB2 (Novocastra, Newcastle, UK) have been previously characterized. A cut-off value of 10% or more positively stained cells out of total cells in ten high-power fields was used in the classification of ER, PR and c-erbB2 expression levels.

### Statistical analysis

The χ^2 ^test (Pearson statistic) was used to determine the differences in clinicopathological features between the two groups of patients. The follow-up duration was calculated from the date of diagnosis until the date of death or last contact. The disease-free survival was the time between diagnosis and confirmation of disease recurrence. The overall survival was the time between diagnosis and death as a result of any cause, regardless of recurrence events. Survival estimates were computed using the Kaplan-Meier method [18] and the differences between survival times were assessed by means of the log rank test [19]. Multivariate analyses were carried out using Cox's proportional hazards model [20]. All statistical analyses were carried out using the SPSS (version 10.0) software package (Chicago, IL, USA).

## Results

A total of 2040 patients were eligible for this study, of which 256 (12.5%) were aged <35 at the time of diagnosis. The median follow-up was 74 months. Histology showed the incidence of medullary carcinoma was significantly higher than ductal carcinoma in the younger group (p = 0.018). There was a significantly higher incidence of nuclear grade 3 in the younger group than in the older group (p = 0.015). Axillary lymph node status, the most prominent prognostic factor in breast cancer, was not significantly different between the two age groups. Also, neither the family history of breast cancer in 1^st ^or 2^nd ^degree relatives, T stage, histological grade, c-erbB2 expression, nor ER or PR status were different between the two groups (Table 1). Frequencies of ER and PR positivity were low, and frequency of c-erbB2 positivity was high, in both age groups compared to frequencies reported in western populations and other Asian studies [21-23].

The proportion of breast-conserving surgery compared to mastectomy was similar in both groups. Axillary lymph node dissection, at least to the first Berg level [24], was performed in 250 (97.7%) younger patients and 1735 (97.3%) older patients. No sentinel lymph node procedure was performed. Adjuvant radiation therapy was administered to patients who underwent breast-conserving surgery and after mastectomy in patients who had four or more positive lymph nodes or a tumor >5 cm in diameter. Adjuvant chemotherapy was administered to 68.0% of younger and 58.7% of older patients. The most common chemotherapy regimen was a combination of cyclophosphamide + methotrexate + 5-FU (CMF) for 6 cycles or anthracycline containing regimen (AC). In terms of hormone therapy, tamoxifen was used for as long as 5 years after completion of surgery and adjuvant therapy. We classified a patient as tamoxifen treatment group if she got tamoxifen through more than a year before recurrence. The proportion of tamoxifen treated patients was significantly lower in young age group. Neither the type of surgery nor the postoperative adjuvant choemotherapy was significantly different between the two age groups (Table 2).

**Figure 1:**
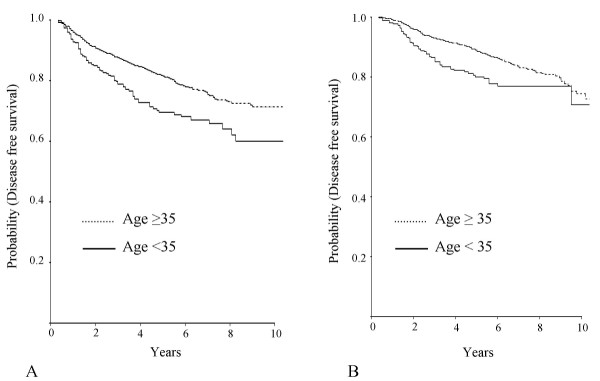
(A) Disease-free survival curves for women <35 vs. ≥ 35 years old. Patients younger than 35 had significantly worse outcomes than their older counterparts (*p *< 0.001). (B) Overall survival curves showing patients younger than 35 had significantly worse outcomes than their older counterparts (*p *= 0.002).

**Figure 2:**
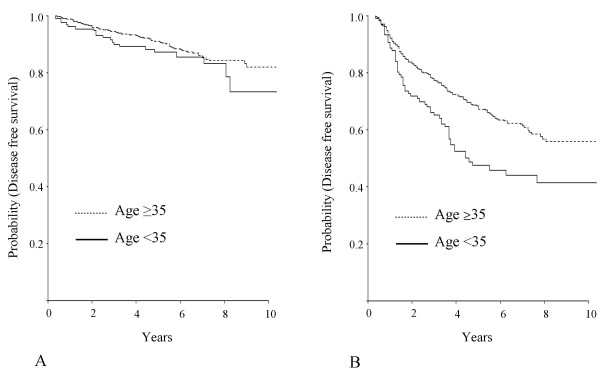
(A) Disease-free survival curves in axillary lymph node-negative patients showing no significant difference between the two age groups (*p *= 0.223). (B) Disease-free survival curves in axillary lymph node-positive patients showing patients younger than 35 had significantly worse outcomes than their older counterparts (*p *< 0.001).

Younger patients had a worse disease free survival (greater probability of recurrence) at all time periods (Fig 1A; *p *< 0.001). At 5 years, the actuarial recurrence rate for patients <35 years old was 30.4% as compared with 18.7% for older patients. This difference persisted at 10 years, at which time the actuarial recurrence rates were 40.1% and 28.6%, respectively. Overall survival among younger patients was significantly worse than for older patients (Fig 1B; *p *= 0.002). The 5-year survival rate was 80.0% for patients aged <35 years as compared with 88.5% for older patients. Stratified analysis according to axillary lymph node status was performed for disease-free survival. In lymph node-negative patients there was no significant difference in disease-free survival between the two age groups (Fig 2A; *p *= 0.223). However, in lymph node-positive patients, disease-free survival was significantly worse in younger patients (Fig 2B; *p *< 0.001).

In multivariate analysis, young age (<35 years) remained a significant predictor of recurrence when entered into a model containing all potential demographic, pathologic and immunohistochemical variables (Table 3. Hazard Ratio (HR), 1.7; 95% confidence interval, 1.1–2.6; *p *= 0.010). However, young age was not a significant independent predictor of overall survival in the same Cox model (table not shown. HR, 1.4; *p *= 0.242). Because hormone therapy was done more frequently in older patients than young age group (Table 2.), we made another multivariate model involving hormone therapy in patients with ER positive and/or PR positive cancer to address the effect of hormone therapy on the prognostic significance of young age. In this analysis, young age was still an independent significant prognostic factor while hormone therapy showed borderline significance (Table 4.).

## Discussion

Our results showed that operable young breast cancer patients (<35 years old) have a worse prognosis than older patients in terms of both overall survival and recurrence. The difference in disease-free survival was clear in patients with axillary lymph node metastasis, but was not observed in lymph node-negative patients. Even after controlling for differences in distribution of potential prognostic factors, young age remained a significant predictor of recurrence.

The present findings support previous reports showing that women diagnosed with breast cancer at a younger age have a poorer prognosis compared with their older counterparts [3-7]. However, those reports suffered from limitations including a small younger patient sample size, a study period spanning too many years during which treatments changed, lack of information about pathological and protein markers, and a heterogeneous case population in terms of ethnicity and treatment strategy.

To our knowledge, the present study is the largest to directly compare the prognosis of younger (<35) breast cancer patients with that of their older counterparts. Moreover, the data in this study were generated from patients of the same ethnicity undergoing treatment at a single institution under the same contemporary strategy of surgery and adjuvant therapy over a relatively short time period (10 years). In addition, this study included a multivariate analysis of the difference in distribution of potential prognostic markers between the two age groups.

The biomarker results in the present study are different to those reported for other populations, including ethnically-related Asian patients. In a study of 1052 Chinese breast cancer patients in Hong Kong, 53% and 61.6% of pre- and postmenopausal women were ER-positive, respectively, and 51.5% and 46.2% were PR-positive, respectively [21]. Those figures are higher than the figures reported in the present study. A recent study of Japanese patients showed 62.2% were ER-positive and only 17.2% were c-erbB2-positive [22]. Merchant et al. found c-erbB2 expression in 30% and 24% of British and Japanese breast cancer patients, respectively [23]. In contrast, Choi et al. reported significant differences in c-erbB2 expression between Korean and white patients (47.5 vs. 15.8%, respectively) using immunohistochemistry and fluorescence in situ hybridization (FISH) techniques. These data suggest c-erbB2 expression may be related to race [25].

Although not a major focus of this study, we found PR and c-erbB2 expression were significant independent predictors of disease recurrence. Currently, the role of PR status as a prognostic factor is not clear, with some evidence to suggest it is useful [26,27] and other evidence to the contrary [28]. As for c-erbB2, its prognostic importance is also controversial. A large number of studies have been published, some reporting positive results and others reporting negative results [29-31]. The prognostic significance of PR and c-erbB2 in this data set can be investigated further as an independent analysis later.

The St. Gallen Consensus Conferences in 1998 and 2001 concluded that age under 35 was a high risk factor for relapse in node-negative breast cancer patients [32,33]. Kroman et al. [34] reported that young women with low-risk breast carcinoma who did not receive adjuvant treatment had a significantly increased risk of death from the disease. Furthermore, Fowble et al. [4] reported that young women with early stage breast cancer, especially those with lymph node-negative disease, had a relatively worse prognosis than older counterpart. In the present study, although no significant difference was observed between the two age groups in lymph node-negative patients, the pattern of survival curves implied younger patients may have a worse prognosis. It may be that a study with a larger case size and a longer follow-up duration would provide enough statistical power to show a significant difference in prognosis for node-negative patients.

It has been suggested that younger women with breast cancer have a poorer prognosis because they present with later stage disease due to either physician or patient delay in diagnosis. However, in this study, no significant difference was found between the two age groups in terms of tumor size or lymph node status. Moreover, multivariate analysis indicated that young age is an independent negative prognostic factor. This issue of delayed diagnosis is not conclusive now and should be elucidated further in subsequent studies.

One possible limitation of this study is that the control group was heterogeneous and contained a mixture of premenopausal and postmenopausal patients. Adami et al. showed complex pattern of survival as a function of age at diagnosis of breast cancer [12]. However, as shown in Table 4, young age remained an independent prognostic factor in multivariate analysis even after patients aged over 50 years were excluded from the control group (*p *= 0.008). Although we did not have full information on each patient's menopausal status at the time of diagnosis, this result suggests that patients under 35 years have even worse prognosis than relatively "less young age" premenopausal patients.

Up to 15–30% of women aged less than 35 years diagnosed with breast cancer are likely to have germ-line BRCA1 or BRCA2 mutations [35,36]. Although we did not investigate BRCA gene mutations in all patients in the present study, we performed BRCA1 and BRCA2 gene mutation scanning in 22 patients who had two or more breast cancer patients in their 1^st ^degree relatives. We found four BRCA2 and one BRCA1 mutations that are thought to be disease-causing (data not shown). Only one of these 5 patients was less than 35 years old at the time of cancer development. It is known that young breast cancer patients are more likely to have an inherited form of the disease [37]. However, the current study showed there was no significant difference in the family history of breast cancer between the two age groups. In the recent report by Choi et al. the prevalence of BRCA1 and BRCA2 mutations in Korean women with breast cancer at a young age (<40) was as high as western population. However, most of the BRCA-associated patients had no family history of breast and/or ovarian cancer. That is, the penetrance appears to be low. They suggested that there may be different genetic and etiologic factors affecting transmission and penetrance of the BRCA genes in Korean patients with breast cancer diagnosed at a young age [38].

Although most current breast cancer investigators agree that young age is an adverse prognostic factor for breast cancer, there have been few studies designed to elucidate the molecular or genetic differences associated with young age breast cancer. Recent CGH (Comparative Genomic Hybridization) analysis suggested that alterations in specific regions on chromosomes might be responsible for the poor outcome of early onset breast cancer [39]. Future research must be focused on this area in order to confirm the characteristics of young age-onset breast cancer at the molecular level.

## Conclusions

These results show that operable young breast cancer patients (<35 years old) have a worse prognosis than older patients in terms of both overall survival and recurrence. Even after controlling for differences in distribution of potential prognostic factors, young age is an independent predictor of recurrence. The underlying biology of young age breast cancer needs to be elucidated and development of tailored treatment for this patient population is crucial.

## Competing interests

The author(s) declare that they have no competing interests.

## Authors' contributions

WH selected cases, reviewed medical records, analyze data, and drafted the manuscript. SWK participated in the data collection and input. IAP carried out the pathological diagnosis and immunohistochemistry. DK performed the statistical analysis. SWK, YKY, SKO, KJC, and DYN conceived of the study, and participated in its design and coordination. All authors read and approved the final manuscript.

## Pre-publication history

The pre-publication history for this paper can be accessed here:



## Figures and Tables

**Table 1:** Clinicopathological characteristics of younger (<35) and older age groups

Characteristics	Age <35 (%) (n = 256)	Age ≥ 35 (%) (n = 1784)	*P *value
Age			
20–25	9 (3.5)		
26–30	56 (21.9)		
31–35	191 (74.6)		
36–40		318 (17.8)	
41–50		791 (44.3)	
51–60		474 (26.6)	
60–70		165 (9.2)	
71-		36 (2.0)	
Family history^a^	22 (8.7)	135 (7.6)	0.511
Histology			
Ductal	238 (93.0)	1624 (91.0)	
Lobular	2 (0.8)	30 (1.7)	
Medullary	10 (3.9)	29 (1.6)	0.018
others	6 (2.3)	101 (5.7)	
T stage			
T1	99 (38.7)	770 (43.2)	
T2	129 (50.4)	855 (47.9)	
T3–4	28 (10.9)	159 (8.9)	0.126
Lymph node metastasis			
Negative	138 (53.9)	1063 (59.6)	
Positive	118 (46.1)	721 (40.4)	0.084
Histological grade			
1–2	78 (58.6)	525 (60.0)	
3	55 (41.4)	350 (40.0)	0.767
Nuclear grade			
1–2	113 (52.6)	901 (61.3)	
3	102 (47.4)	570 (38.7)	0.015
ER			
Positive	97 (47.1)	907 (51.8)	
Negative	109 (52.9)	843 (48.2)	0.198
PR			
Positive	73 (36.7)	708 (43.5)	
Negative	126 (63.3)	921 (56.5)	0.068
c-erbB2			
Negative	94 (52.8)	647 (46.4)	
Positive	84 (47.2)	748 (53.6)	0.106

^a^Patients who have 1st or 2nd degree relatives with breast cancer

**Table 2:** Treatment characteristics

Characteristics	Age <35 (%)	Age ≥ 35 (%)	*P *value
Surgery			
Mastectomy	211 (82.4)	1482 (83.1)	0.796
Conservation	45 (17.6)	302 (16.9)	
Chemotherapy			
Lymph node (-)	65/138 (47.1)	421/1063 (39.6)	0.281
Lymph node (+)	109/118 (92.4)	627/721 (87.0)	0.674
Radiation therapy	73 (28.5)	462 (25.9)	0.373
Hormone therapy	54 (21.1)	492 (27.6)	0.028

**Table 3:** Multivariate analysis for predictors of recurrence based on the Cox proportional hazards regression model

Variables	HR	95% Confidence interval	*p*
Age <35 years	1.7	1.14–2.61	0.010
Tumor size ≥ 2 cm	2.0	1.30–3.07	0.002
Lymph node-positive	3.8	2.64–5.67	<0.001
Nuclear grade 3	1.4	0.90–2.39	0.124
Histological grade 3	0.9	0.54–1.49	0.675
ER	1.1	0.77–1.63	0.549
PR	2.1	1.41–3.19	0.001
c-erbB_2_	1.4	1.04–2.05	0.030

**Table 4:** Multivariate analysis for predictors of recurrence involving hormone therapy in ER(+) and/or PR(+) patients

Variables	HR	95% Confidence interval	*p*
Age <35 years	2.1	1.14–4.20	0.018
Tumor size ≥ 2 cm	2.2	1.39–3.62	0.001
Lymph node-positive	3.3	1.81–6.14	<0.001
Nuclear grade 3	1.0	0.45–2.31	0.961
Histological grade 3	1.2	0.55–2.85	0.576
Hormone therapy (yes)	1.6	0.93–2.77	0.086
c-erbB_2_	1.4	0.81–2.42	0.228

**Table 5:** Multivariate analysis for predictors of recurrence based on the Cox proportional hazards regression model after exclusion of patients >50 years old.

Variables	HR	95% Confidence interval	*p*
Age <35 years	1.8	1.17–2.81	0.008
Tumor size ≥ 2 cm	1.7	1.06–2.84	0.028
Lymph node-positive	4.2	2.64–6.82	<0.001
Nuclear grade 3	1.0	0.60–1.91	0.810
Histological grade 3	1.0	0.58–1.89	0.883
ER	1.1	0.71–1.78	0.625
PR	2.0	1.27–3.37	0.004
c-erbB_2_	1.4	0.97–2.21	0.069
